# Effect of Pycnogenol Supplementation on Blood Pressure: A Systematic Review and Meta-analysis

**Published:** 2018-06

**Authors:** Zheng ZHANG, Xing TONG, Yu-Lu WEI, Lin ZHAO, Jia-Ying XU, Li-Qiang QIN

**Affiliations:** 1. Dept. of Nutrition and Food Hygiene, School of Public Health, Soochow University, Suzhou, China; 2. Collaborative Innovation Center of Radiological Medicine of Jiangsu Higher Education Institutions, School of Radiation Medicine and Protection, Soochow University, Suzhou, China

**Keywords:** Pycnogenol, Blood pressure, Randomized controlled trial, Meta-analysis

## Abstract

**Background:**

Pycnogenol exhibits many biological activities, including control of blood pressure (BP). However, the reported results are inconsistent because of varied characteristics of participants and quality of studies. Thus, a meta-analysis was conducted to examine the effect of Pycnogenol supplementation on BP.

**Methods:**

This literature search of PubMed, the Web of Science and the Cochrane library was performed in May 2016 to identify eligible studies. Reference lists of the retrieved articles were also reviewed. Either a fixed-effects or, in the presence of heterogeneity, a random-effects model was used to calculate the effect of combined treatment.

**Results:**

We identified nine trials involving 549 participants who received Pycnogenol supplementation ranging from 150 mg/d to 200 mg/d. Compared with the control, the pooled estimate of change in systolic and diastolic BPs were −3.22 mmHg (95% CI: −6.20, −0.24) and −3.11 mmHg (95% CI: −4.60, −1.62), respectively. Subgroup analyses showed higher BP reduction among hypertensive participants or those who received intervention for more than 12 wk. However, this significant reduction was not observed in well-designed trials.

**Conclusion:**

This meta-analysis with nine trials provides better evidence that Pycnogenol exerts beneficial effects on BP.

## Introduction

People have shown keen interest in herbal medicine with the hope that they can improve their health condition through diet or consumption of natural compound. Pycnogenol is a nutritional supplement used as a phytochemical remedy worldwide (1). The term Pycnogenol was intended to serve as a scientific name for this class of polyphenols (2); however, this term essentially refers to a specific blend of procyanidins extracted from a French maritime pine bark. Pycnogenol is standardized to 70%±5% procyanidins; the extract also contains catechin, taxifolin, and a range of phenolic acids, represented by cinnamic acid and benzoic acid derivatives (1).

Pycnogenol supplementation produces various potentially protective effects against chronic diseases, such as metabolic syndrome, obesity, dyslipidemia, diabetes and hypertension (3). We particularly focus on its effect on blood pressure (BP). An early animal study found that systolic blood pressure (SBP) and diastolic blood pressure (DBP) decreased in a dose-dependent manner after intravenous administration of pine bark extract to SD rats (4). A long-term animal study reported a slight but significant SBP reduction in spontaneously hypertensive rats treated with Pycnogenol for 6 wk (5). In fact, the effect of Pycnogenol on human BP has gained increased research attention. A randomized controlled trial (RCT) showed that oral administration of Pycnogenol reduced SBP to the normal value in hypertensive patients (6). A subsequent RCT indicated that Pycnogenol supplementation in hypertensive patients reduced the need for nifedipine, a calcium antagonist used as a coronary vasodilator (7). However, the sum of BP deceased by 1.0 mmHg in Pycnogenol-treated group and even by 1.9 mmHg in placebo group after a 12-wk intervention (8). The discrepancies in BP-lowering effect are mainly attributed to inter-study variations in terms of inclusion criteria, trial design, supplemental dosage, and duration of intervention.

Therefore, we conducted a meta-analysis to examine whether or not Pycnogenol supplementation is beneficial in lowering BP and investigate the potential sources of heterogeneity across studies.

## Methods

### Search strategy and study selection

We follow the Preferred Reporting Items for Systematic Reviews and Meta-Analysis (PRISMA) guidelines in the report of this meta-analysis (9). We conducted a systematic literature search of PubMed, the Web of Science and the Cochrane library through May 2016, using the following search terms: “pycnogenol OR maritime pine bark OR proanthocyanidin” in combination with “blood pressure OR hypertension OR endothelial OR flow-mediated dilation OR vascular”. No restrictions were imposed. Reference lists were also reviewed. We did not contact the authors of the primary studies for additional information. We also did not try to consider the unpublished studies. Trials were included in the analysis if they were RCT and clearly reported the dosage of Pycnogenol supplementation, intervention duration and BP levels before and after the trials. Studies in Pycnogenol combined with drug treatment included if the control group was also treated. If more than one time point for the follow up was reported, the data from the longest period were used. Likewise, the data from the highest dose were used when more than one dose was administered for supplementation. In the case of multiple publications with duplicate/overlapped data for the same trial, the article with more detailed information was selected.

### Data extraction and quality assessment

We recorded the following characteristics of each study: first author’s name, publication year, and study design, sample size, study period, daily dose of Pycnogenol, intervention period. We also extracted the following participant characteristics: gender, mean age, health condition, baseline BP and change in BP of each study. The Jadad score, a scale that ranges from 0 to 5 according to the descriptions of randomization, blinding and reporting of participant withdrawals, was used to measure the quality of each study (10).

### Data synthesis and analysis

For parallel trials, the net changes in each outcome in the intervention and control groups were reported as differences between mean values before and after treatments. For crossover trials, net changes were calculated as differences in the post treatment values of each group. Cohen method was used to combine SD. Studies with no reported SD values had their values imputed from standard errors, the confidence interval (CI) or *P*-values using a standard formula. If only SD for the baseline and final values were provided, SD for the net changes were imputed according to the method of Follmann using a correlation coefficient of 0.5 (11).

The heterogeneity between the studies was tested using the Cochran’s Q test at the *P*<0.10 level of significance and quantified by the *I*^2^ statistic, which describes the inconsistency across studies (12). In the presence of significant heterogeneity, the random-effects model was used to calculate the pooled effect size, otherwise, the fixed-effects model was applied (13). To explore the possible influences of study design and participants characteristics, we further conducted pre-specified subgroup analysis stratified by study design (double blind *vs.* non-double blind design; parallel *vs.* cross-over design), hypertension status, Jadad score, and duration of supplementation. We also performed a sensitivity analysis, in which a single trial was omitted each time and the effect size was recalculated to investigate its influence on the overall effect size. Potential publication bias was assessed using Begg’s funnel plots and Egger’s regression test at the *P*<0.10 level of significance (14).

All analyses were conducted by using STATA version 14.0 (StataCorp, College Station, TX, USA). *P*<0.05 was considered statistically significant, except where otherwise specified.

## Results

### Search results

**Fig. 1:**
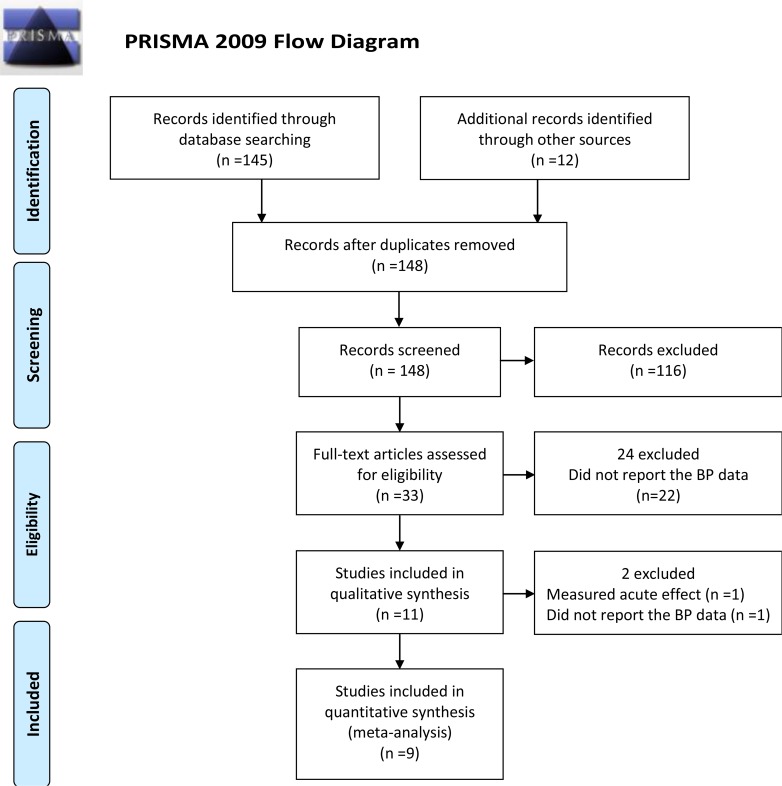
Flow chart of study selection

We initially found 148 articles, the majority of excluded based on their title and abstract. After reviewing the full text of the remaining 33 studies, 22 studies were excluded because they did not record BP at the baseline or after intervention. Among the remaining 11 potentially relevant articles, two studies were excluded because they measured an acute effect or they did not include a control group. Finally, Nine trials were included in our meta-analysis (6, 8, 15–21) (Fig. 1).

### Study characteristics

Table 1 shows the characteristics of the included trials. These trials were published from 2001 to 2015; 5 were conducted in Italy, 2 in USA, and 1 each in Switzerland and Japan. A parallel design was used in seven trials and a cross-over design was used in the two other trials. Five trials employed double-blind method, one trial was an open label-study, and three trials did not mention anything about blinding. Sample sizes varied from 16 to 130 with 276 participants in the supplemental groups and 273 in the control groups. The mean age varied from 22.4 yr old to 63.1 yr old. Except in Nishioka’s trial, which involved health young men, the other trials evaluated patients with borderline hypertension, hypertension, metabolic syndrome, type 2 diabetes mellitus (T2DM), or coronary artery disease. A drug against hypertension was used in two trials (18, 19) and a drug against diabetes was used in one trial (21). These drugs were used both in the intervention and control groups. The amounts of Pycnogenol were 150 (five trials), 180 (one trial) and 200 (three trials) mg/d. No other nutritional elements, vitamins, or drugs were used. However, some trials required participants to receive a dietary education or to follow the guidelines of healthy lifestyle during the observation period (15, 16, 18). The Jadad score of these trials was relatively low, and only four trials had a score of not lower than 3.

**Table 1:** Characteristic of the trials and participants in this meta-analysis

***Author (yr)***	***Country***	***Design***	***Sample size (intervention/control)***	***Health status***	***Sex (M/F)***	***Age (yr)***	***Baselin BP (mmHg)***		***Daily Dose (mg)***	***Duration (week)***	***Jadad score***

							Intervention	Control			
Hu (2015)	Italy	P	16/16	Borderline hypertension	18/4	44.5	132.2/84.3	134.3/85	150	12	1
Belcaro (2013)	Italy	P	64/66	Metabolic syndrome	64/66	45.5	144/87.6	143.2/87.2	150	25	2
Enseleit (2012)	Switzerland	X, DB	23/23	Coronary artery disease	19/4	63.1	125.8/75.0	124.8/73.9	200	8	4
Cesarone (2010)	Italy	P	29/26	Hypertension	34/21	53.7	188/96.3	186/96	150	25	2
Drieling (2010)	USA	P, DB	64/66	Metabolic syndrome	82/48	55	132.6/78.6	133.2/79.9	200	12	5
Stuard (2010)	Italy	P, O	31/27	Metabolic syndrome	31/27	58.7	189.3/97.2	188.8/95.2	150	25	2
Nishioka (2007)	Japan	P, DB	8/8	Health	16/0	22.4	114.2/62.2	115.6/64.3	180	2	4
Cesarone (2006)	Italy	P, DB	30/30	Diabetes	34/26	59	131(4)/88(3)	133/85	150	4	2
Hosseini (2001)	USA	X, DB	11/11	Hypertension	14/8	50.3	139.4/93.4	139.9/93.8	200	8	3

P: parallel; X: cross-over; O: open; DB: double blind

### Effect of Pycnogenol supplementation on BP

**Fig. 2:**
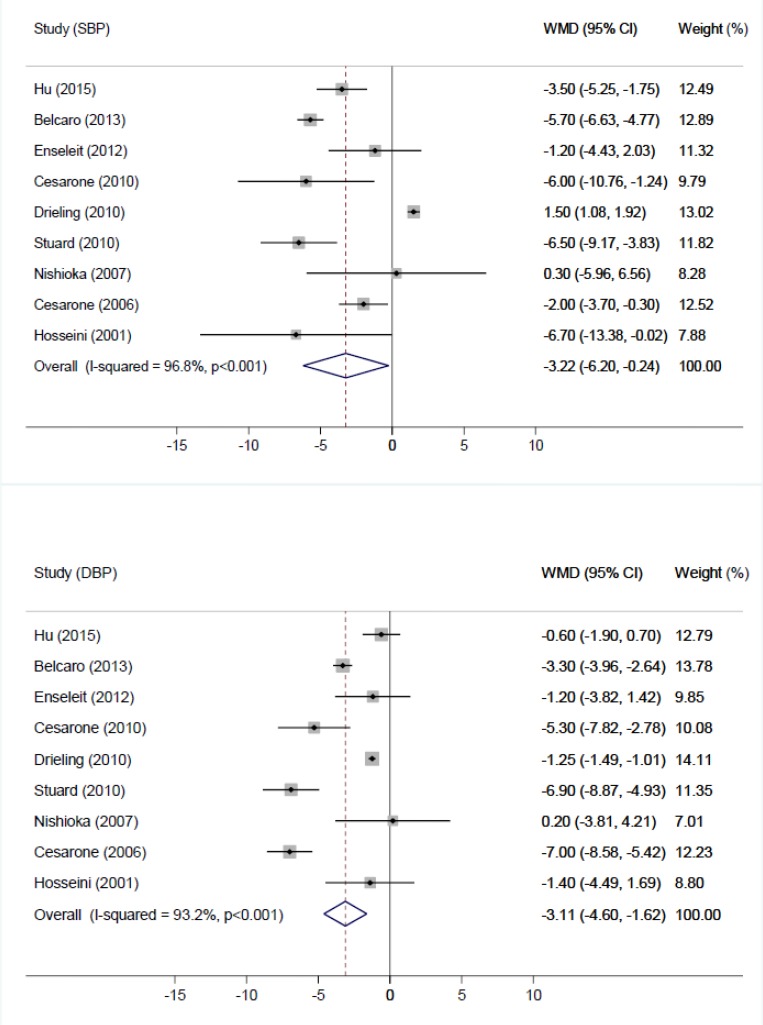
Pooled estimates of Pycnogenol supplementation on systolic blood pressure (SBP) and diastolic blood pressure (DBP). WMD, weighted mean difference

Compared with the control group, the intervention group was associated with an average net change in BP ranging from −6.70 to 1.50 mmHg for SBP and −7.00 to 0.20 mmHg for DBP. A trend toward intervention-related reduction in SBP was observed in seven trials, with six trials showing a significant reduction. In addition, a trend toward intervention-related reduction in DBP was observed in eight trials, with a significant reduction in five trials. The tests for heterogeneity indicated that the supplemental effect significantly varied across studies (*P*<0.001 for SBP and DBP), and *I*^2^ values were 96.8% for SBP and 93.2% for DBP. Thus, the random-effects model was used. The overall pooled estimates of the effect of Pycnogenol were −3.22 mmHg (95% CI −6.20, −0.24; *P*=0.034) for SBP and −3.11 mmHg (95% CI −4.60, −1.62; *P*<0.01) for DBP (Fig. 2).

### Subgroup and sensitivity analyses

Table 2 shows the results of the subgroup analyses. When trials were stratified according to study design, the effect of supplementation on BP was not observed in trials with a double-blind design. Pycnogenol supplementation did not affect SBP when trials were stratified by parallel and crossover design. However, the effect of supplementation on DBP was observed in trials with a parallel design. No effect of Pycnogenol supplementation on SBP was observed in trials with Jadad scores ≥3.

**Table 2:** Subgroup analyses according to study design and participants’ characteristics

***Groups***	***N***	***SBP***				***DBP***			
		**Net Change (95% CI)**	***P*^1^**	***I*^2^ (%)**	***P*^2^**	**Net Change (95% CI)**	***P*^1^**	***I*^2^ (%)**	***P*^2^**
Total	9	−3.22 (−6.20, −0.24)	0.034	96.8	<0.001	−3.28 (−5.26, −1.30)	<0.001	95.7	<0.001
Study design									
DB	5	−0.97 (−3.52, 1.58)	0.457	82.7	<0.001	−2.31 (−5.12, 0.51)	0.109	92.1	<0.001
Non-DB	4	−5.22 (−6.59, −3.85)	<0.001	46.3	50.133	−3.87 (−6.17, −1.57)	<0.001	90.5	<0.001
Parallel	7	−3.17 (−6.55, 0.22)	0.067	97.5	<0.001	−3.53 (−5.24, −1.82)	<0.001	94.9	<0.001
Cross-over	2	−3.14 (−8.29, 2.01)	0.232	52.6	0.146	−1.28 (−3.28, 0.72)	0.208	0	0.923
BaselineBP(mmHg)									
SBP<140 or DBP<90	5	−1.07 (−3.73, 1.59)	0.430	91.0	<0.001	−2.14 (−4.39, 0.11)	0.062	92.3	<0.001
SBP≥140 or DBP≥90	4	−5.81 (−6.66, −4.95)	<0.001	0	0.943	−4.31 (−6.43, −2.20)	0.002	80.2	0.002
Jadad Score									
≥3	4	−0.54 (−3.46, 2.38)	0.718	64.6	0.037	−1.25 (−1.48, −1.01)	<0.001	0	0.916
<3	5	−4.52 (−6.33, −2.71)	<0.001	77.7	0.001	−4.53 (−6.78, −2.28)	<0.001	92.2	<0.001
Duration									
≤12 week	6	−1.58 (−4.20, 1.03)	0.234	89.9	<0.001	−2.04 (−4.04, −0.05)	0.044	90.4	<0.001
>12 week	3	−5.79 (−6.66, −4.93)	<0.001	0	0.854	−4.91 (−6.63, −3.19)	<0.001	58.5	0.090

*P*^1^ value of subgroup analysis via Z-test, *P*^2^ value for heterogeneity

DB: double blind; SBP: systolic blood pressure; DBP: diastolic blood pressure

With regard to baseline BP, BP was significantly reduced in hypertensive participants displaying an SBP of ≥140 or DBP of ≥90, but not in their counterparts. SBP was also significantly reduced only among trials wherein the intervention duration was >12 wk, and DBP reduction tended to be greater in these trials. Subgroup analyses according to mean age and daily dose were not performed because of narrow ranges.

Analysis examining the influence of an individual trial on the overall effect size by omitting one trial at each turn yielded a range feom −2.78 mmHg (95% CI: −5.91, 0.35, *P*=0.08) to −3.98 mmHg (95% CI: −5.65, −2.30, *P*<0.01) for SBP and from −2.64 mmHg (95% CI: −4.09, −1.18, *P*<0.01) to −3.46 mmHg (95% CI: −5.15, −1.77, *P*<0.01) for DBP. The pooled estimate of Pycnogenol on SBP became insignificant after excluding the studies of Stuar (*P*=0.08), Cesarone (*P*=0.06), or Hu (*P*=0.06). Unfortunately, heterogeneity still existed when any trial was omitted.

### Publication bias

**Fig. 3:**
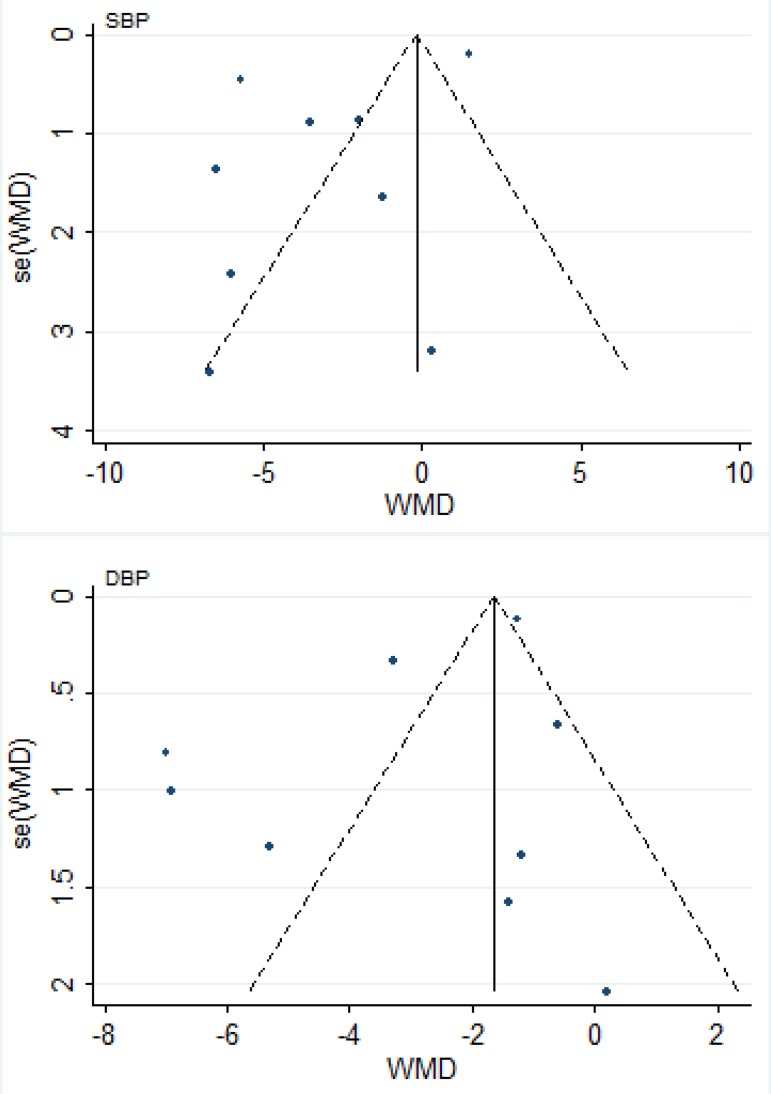
Funnel plot of Pycnogenol supplementation on systolic blood pressure (SBP) and diastolic blood pressure (DBP). WMD, weighted mean difference

Visual inspection of the funnel plots showed some asymmetry (Fig. 3). However, Results from Begg’s and Egger’s tests also did not indicate the evidence of publication bias (SBP: Begg *P*=0.60, Egger *P*=0.12; DBP: Begg *P*=0.92, Egger *P*=0.14).

## Discussion

This meta-analysis is the first to report that Pycnogenol supplementation significantly reduced SBP and DBP by approximately 3 mmHg. This BP-lowering effect was also supported by two observational studies, which provided the BP control rate. In a trial, 100 mg of Pycnogenol was administered in hypertensive patients for 12 wk. As a result, 15 mg of nifedipine was sufficient to lower the BP to normal value compared with use of 21.5 mg in the control group (7). In another study that includes T2DM patients receiving pharmaceutical treatment showed that 58.3% of the Pycnogenol-treated subjects achieved BP control at the end of 12 wk. However, only 20.8% of the subjects maintained control in the control group (22). A considerable normalization of SBP and DBP was reported after 2 months of supplementation of OPC-3 in subjects with metabolic syndrome, whereas minimal changes were found in the control group (23).

Although the precise mechanisms were not fully understood, the BP-lowering effect of Pycnogenol involves angiotensin converting enzyme (ACE) inhibition, nitric oxide (NO) production, and antioxidation and anti-inflammatory activities. Pine bark extract exerted inhibitory effect on ACE with concentration of 50% inhibition as 34.7μg/ml (4). Two trials included in this meta-analysis evaluated the effects of Pycnogenol as an adjunct to ACE-inhibitor ramipril for treatment of hypertensive patients. Administration of ramipril plus Pycnogenol exerted a significantly greater effects on BP than that of ramipril alone (18,19). ACE inhibitor should reduce serum angiotensin-II level and improve flow-mediated vasodilation. However, plasma level of angiotensin II was not lowered to a considerable extent in Pycnogenol group, compared with that in the control group (7). Thus, further studies required to investigate Pycnogenol as an ACE inhibitor in the clinically relevant action.

On the other hand, Pycnogenol enhances the endothelial production of NO through the enzyme nitric oxide synthase (NOS). An in vitro study showed that Pycnogenol relaxes the adrenaline-induced contractions in the aortic blood vessels of rat. This response was due to enhance NO levels because the NOS inhibitor reverses the relaxation, and this response in turn is reversed by addition of L-arginine, the normal substrate for NOS (24). In Nishioka’s trial, Pycnogenol supplementation for 2 wk significantly augmented the response of forearm blood flow to acetylcholine, an endothelium-dependent vasodilator acetylcholine. Interestingly, administration of NOS inhibitor completely abolished this response, suggesting that Pycnogenol plays a role by increasing NO production (20). Oxidative stress is important in the development and maintenance of hypertension. Belcaro directly quantified reactive oxygen metabolites by using the free radical analytical system in patients with metabolic syndrome and found that reduction in oxidative stress was significantly more pronounced after 6 months in the Pycnogenol supplementation group than that in the control group (16). In another study, healthy subjects received Pycnogenol for 6 wk and their plasma oxygen radical absorbance capacity significantly increased. Interestingly, this antioxidant activity returned to the baseline value after a 4-wk washout period (25). In addition to its antioxidant activity, Pycnogenol demonstrated an anti-inflammatory activity. C-reactive protein (CRP), the most widely known inflammatory factor, is associated with vascular stiffness, BP, and atherosclerosis (26). In an RCT involving patients with osteoarthritis, Pycnogenol supplementation for 3 months significantly reduced plasma CRP compared with that in the control group; in addition, Pycnogenol reduced plasma free radicals (27). OPC-3 supplementation for 2 months also dramatically lowered plasma CRP, but exerted minor effects in the control group (23).

The results of subgroup analysis indicated that BP-lowering effect was observed among hypertensive patients in the trials. Thus, participants with higher baseline BP, who mostly needed treatment, were more likely to benefit from Pycnogenol supplementation. On the other hand, BP reduction was more pronounced in trials with intervention duration of more than 12 wk. Therefore, a longer period is required to improve BP condition. However, subgroup analysis did not reveal BP-lowering effect among trials with double-blind design and cross-over design, and SBP-lowering effect among trials with Jadad scores ≥3. Open label and the lack of clear data collection techniques resulted in the low Jadad scores in these trials. The lack of rigorous RCT design was the main limitation of this meta-analysis.

In addition to individual design, this meta-analysis was limited by a considerable heterogeneity across studies. In term of characteristics of participants, the trials involved patients with hypertension, metabolic syndrome, diabetes, and healthy subjects. Healthy status may differently influence the effect of Pycnogenol on BP response. In addition, genetic background or a gene-diet interaction could be the sources of heterogeneity across studies. BP decrease diversely responses to ACE inhibitor between Whites and Blacks (28). Inclusion of various races in Drieling’s trial was possibly resulted in failure to observe the effect of the supplementation (8). In term of intervention, we did not perform subgroup analysis according to supplemental dosage because of the narrow range between 150 and 200 mg per day. Moreover, Pycnogenol is not easily standardized and mixed with monomer, dimer, and trimer chemical components obtained from pine bark extracts (29). This phenomenon may account for variations in physiologic effects among different trials. Furthermore, some trials tested Pycnogenol as an adjunct to conventional pharmacologic treatment and some trials required the participants to receive a healthy lifestyle education; both have probably masked the effects of Pycnogenol on BP. In term of outcome, not all trials were originally designed to investigate the BP-modulating properties of Pycnogenol.

## Conclusion

The findings demonstrated the favorable effects of Pycnogenol supplementation on BP reductions especially among hypertensive participants. However, the biological significance of findings should be interpreted with caution because of the heterogeneity and low-quality design of individual trials.

## Ethical considerations

Ethical issues (Including plagiarism, informed consent, misconduct, data fabrication and/or falsification, double publication and/or submission, redundancy, etc.) have been completely observed by the authors.
